# Perceived cause and determinants of help-seeking behavior of schizophrenia among Gondar Zuria district residents, Northwest Ethiopia

**DOI:** 10.1016/j.heliyon.2021.e07212

**Published:** 2021-06-04

**Authors:** Enyew Getaneh Mekonen, Ayenew Kassie Tesema, Belayneh Shetie Workneh, Maereg Wolde, Niguse Yigzaw Muluneh

**Affiliations:** a University of Gondar, College of Medicine and Health Sciences, School of Nursing, Department of Surgical Nursing, Gondar, Ethiopia; b University of Gondar, College of Medicine and Health Sciences, Institute of Public Health, Department of Health Education and Behavioral Sciences, Gondar, Ethiopia; c University of Gondar, College of Medicine and Health Sciences, School of Nursing, Department of Emergency and Critical Care Nursing, Gondar, Ethiopia; d University of Gondar, College of Medicine and Health Sciences, Department of Psychiatry, Gondar, Ethiopia

**Keywords:** Schizophrenia, Perceived cause, Help-seeking behavior, Gondar Zuria district

## Abstract

**Introduction:**

The cause of schizophrenia could be a genetic predisposition, environment, viral infections, exposure to poison substances, living in a highly-populated area, and prenatal exposure to hunger mainly in the first three months. Evidence showed that the perceived cause of schizophrenia is supernatural, biological, spiritual, and social causes. Studies in Ethiopia showed that most of the general population perceived the causes of schizophrenia as traditional and the help they seek ranges to medical, religious, and social.

**Objective:**

This study aimed to assess perceived cause and determinants of help-seeking behavior of schizophrenia among Gondar Zuria district residents, 2020

**Methods:**

A community-based cross-sectional study was conducted from December 3 to 25, 2020. A simple random sampling technique was employed to select 435 study participants. Data were collected through a face-to-face interview, entered into EPI DATA version 3, and analyzed using SPSS version 21. Bivariable and multivariable binary logistic regression analyses were employed to identify factors significantly associated with help-seeking behavior for schizophrenia. Statistical significance was declared at p-value < 0.05 with 95% confidence interval.

**Results:**

Nearly two-thirds (63.8%), the majority (90.8%), and more than half (52.5%) of the participants seek medical, religious, and social help for schizophrenia respectively. Being student (AOR = 3.43; 95% CI: 1.44, 8.15), unemployed (AOR = 4.87; 95% CI: 1.4, 16.40), perceived biological cause (AOR = 1.7; 95% CI: 1.01, 2.89), perceived religious cause (AOR = 0.48; 95% CI: 0.29, 0.80), and perceived social cause (AOR = 2.05; 95% CI: 1.29, 3.25) were significantly associated with medical help seeking. Attending primary school (AOR = 0.17; 95% CI: 0.04, 0.76), employed (AOR = 0.12; 95% CI: 0.02, 0.64), perceived religious cause (AOR = 2.34; 95% CI: 1.06, 5.11) were significantly associated with religious help. Being in the age group of 18–24 years (AOR = 3.5; 95% CI: 1.33, 9.18) and 25–44 years (AOR = 1.94; 95% CI: 1.03, 3.68) were significantly associated with social intervention.

**Conclusion:**

Nearly two-thirds, the majority, and more than half of the respondents seek medical, religious, and social help for schizophrenia respectively. Being student, unemployed, perceived biological case, and perceived social cause increases the odds of seeking medical help while perceived religious cause decreases it. Being unemployed, attending primary school decreases the odds of seeking religious help whereas perceived religious cause increases it. Young adults have higher odds of social help-seeking behavior. It is better to create awareness for the community and consider integrating religious and social interventions into medical interventions.

## 1 Introduction

Schizophrenia is a complex mental disorder, with a heterogeneous combination of symptoms, typical onset in late adolescence or early adulthood [1]. The cause of schizophrenia could be a genetic predisposition, environment, viral infections such as herpes zoster, exposure to poison substances such as lead, prenatal exposure to hunger mainly in the first three months, and living in a highly-populated area [2]. Although schizophrenia by itself is not fatal, people with schizophrenia are twice at risk of death as the general population since it has various adverse consequences like social disability, social stigma, and social cost [3].

The general population in different countries had a different perception of schizophrenia. However, people living in western countries focus mainly on biological such as genetics, disease of the brain, and infection, and social risk factors such as personal weakness [4, 5, 6]. A study conducted in the united kingdom showed that emotional state could cause schizophrenia [7]. A similar study conducted in China showed that 84% of participants perceived the cause of schizophrenia to social, interpersonal, and psychological problems [8], and a study in Malaysia showed that lack of social support, a chemical imbalance in the Brian, and believes in supernatural factors were perceived by the participants as a cause of schizophrenia [9].

A study conducted in Pakistan revealed that individuals who perceive a biological cause for the disease were almost 13 times more likely to seek medical help for a relative with schizophrenia than those who gave other reasons for the disease [4]. A study in Cambodia revealed the community didn't first seek help medical care and Nigerian studies show that most of the participants had visited traditional/faith healers as the first treatment [9, 10, 11, 12].

Studies in Africa revealed the perceived cause of schizophrenia among different cultures. A study in Ghana showed 94% perceived the cause is due to witchcraft/evil spirits, and 66% felt that it is due to divine punishment [13]. In Ethiopia, people perceive the cause of schizophrenia as religious [14], misuse of the drug [15], poverty [16], and punishment for sins/wrongdoings [17].

Studies showed that individuals seek religious help for schizophrenia. A study in Turkey showed 74% [18], in china 30.8% [19] pursued non-medical options (i.e., relatives, praying to Buddha), in Pakistan religious remedies (19.3%) [5], sought help from religious sources for their illness. Medical help was also the first choice of their illness as indicated in Vietnam [20], about 74% of participants sought medical help in china [21]. Different studies showed that demographic factors such as age, gender, residence, and educational level of the participants were indicated as determinant factors of help-seeking behavior of schizophrenia [4, 5, 17]. In Ethiopia, little is known about the perceived causes and determinants of help-seeking behavior of schizophrenia, and no study is conducted in the Gondar Zuria district specifically. Knowing the community's perceived cause of schizophrenia and help-seeking behavior is important to design tailored interventions to change their attitude and behavior. Thus, it helps to increase medical help-seeking earlier. The finding of the study may help health policymakers and health professionals in developing a new approach for increasing the help-seeking behavior of schizophrenic individuals. Therefore, this study aimed to assess the perceived cause and determinants of help-seeking behavior of schizophrenia among Gondar Zuria district adults.

## 2 Objectives of the study

To assess the perceived cause of schizophrenia among Gondar zuria district residents.

To assess the help-seeking behavior of schizophrenia among Gondar zuria district residents.

To identify the determinant factors of help-seeking behavior of schizophrenia among Gondar zuria district residents.

## 3 Methods and materials

### 3.1 Study design and period

A community-based cross-sectional study was conducted from December 3 to 25, 2020.

### 3.2 Study setting

The study was conducted at Gondar Zuria district, Northwest Ethiopia. Gondar Zuria district is one of the woredas in the central Gondar zone. Maksegnit is the town for the Gondar Zuria district. It has 2 Kebele, which encompasses about 8,136 females and 7, 816 males. The district has only one health center and it doesn't have any other health care services. Any mental illness cases including schizophrenia are referred to the University of Gondar comprehensive specialized referral hospital.

### 3.3 Study participants

All individuals who were above 18 years old age and live for at least six months in Maksegnit town were included in the study. Individuals who were seriously ill and unable to respond to the questions were excluded from the study.

### 3.4 Sample size determination

The sample size for the first objective was calculated using the single proportion population formula by taking the proportion of traditional perception of schizophrenia (73.7%), proportion of help-seeking behavior (50%), 95% confidence interval, and 5% margin of error. For the second objective, it was calculated by using Epi Info software version 7 (a public domain software package designed for the global public health community of practitioners and researchers which is used for data entry, analysis, and sample size calculation) by considering the following assumptions: 95% confidence interval, 80% power, 50% proportion of help-seeking behavior, and 73.7% proportion of traditional perception of schizophrenia. The sample size calculated with the factors was less than the sample size calculated by the first objective on the help-seeking behavior and the perceived causes of schizophrenia, which was the largest sample size. Therefore, the final sample size for this study was 435.

### 3.5 Sampling procedure

There are two Kebele in Maksegnit town and the study was conducted in both Kebeles. Households within each Kebele were selected by a systematic sampling technique while selecting the eligible respondents from the selected household simple random sampling technique was applied.

### 3.6 Study variables

The dependent variables of this study were help-seeking behaviors (medical, religious, and social help). The independent variables of this study were socio-demographic characteristics (age, sex, educational status, occupation, and marital status) and perceived causes (biological, psychosocial stressors, social, religious).

### 3.7 Data collection and measurements

Data were collected through a face-to-face interview by trained data collectors. The Causal Models Questionnaire for Schizophrenia (CMQS) as well as questionnaires adapted from previous studies to assess socio-demographic characteristics and other associated factors were used for data collection. The CMQS has 36 items to be asked whether individuals perceived each item as a possible cause of schizophrenia or not. It has been used in different studies to assess the perceived causes of schizophrenia with good validity and reliability [5, 17, 22]. A case vignette was prepared by the investigators based on the proper DSM-V diagnostic criteria of schizophrenia and commented on by senior psychiatrists in the University of Gondar Comprehensive Specialized Referral Hospital to identify the perceived cause of schizophrenia. Four trained diploma nurses interviewed the community and were supervised in the field during data collection.

### 3.8 Data processing and analysis

Data clean-up and cross-checking were done before analysis. Checked, cleaned, and coded data were entered into EPI DATA version 3 (a program for data entry and documentation of data) and exported to SPSS version 21 (a statistical software used by researchers to perform statistical analysis) for analysis. Descriptive statistics like frequencies, percentages, mean, and standard deviation were used to present data. Binary logistic regression was used to see associated factors with the dependent variable. Those variables with a P value less than 0.2 were entered into multivariable analysis to identify the independent associated factors and confounders were controlled. Finally, Variables with a p-value < 0.05 were considered as significantly associated.

### 3.9 Data quality control

The tool was first developed in the English language and translated to the Amharic (Local language) with back translation to English for consistency. The data collection tool was reviewed by four experts (two psychiatrists and two statisticians). To assure the quality of data the questionnaire was pretested two weeks before the actual data collection time by using 5% of the total sample size. Modifications on the instrument, like unclear questions and ambiguous words, were made accordingly. The resultant data were also used to calculate Cronbach's alpha, which was 0.82. Training about the data collection procedure was given to data collectors and supervised by principal investigators on the actual data collection site. The collected data were reviewed and checked for completeness before data entry.

### 3.10 Ethics approval and informed consent

Ethical clearance was obtained from the institutional review board of the University of Gondar. A written letter of permission was submitted to the Gondar Zuria district administration office. The study participants were provided with clear information about the purpose of the study and asked if they were willing to participate in the study. Data were collected after receiving verbal consent from those who were willing to participate in the study. The anonymity of the respondents was preserved and their information was kept secret and not disclosed to anyone except for the study.

## 4 Results

### 4.1 Socio-demographic characteristics of the respondents

A total of 423 residents participated in this study, with a response rate of 97.2%. Nearly two-thirds (63.6%) of the respondents were male and more than half (53.2%) of the participants were married. The mean (±SD) age of the respondents was 31.8 ± 13.1 years. Regarding the level of education, about 26.2% of the respondents were completed secondary school. The majority (78.7%) of the respondents were orthodox Christian in their religion. Regarding their occupation, 30.3% of the participants were students (Table 1).

**Table 1:** Socio-demographic characteristics of Gondar Zuria district residents, Northwest Ethiopia, 2021 (n = 423).

Variables	Category	Frequency (n = 423)	Percentage (100%)
Age	18–24 25–44 45 and above	150 202 71	35.5 47.8 16.8
Sex	Female Male	154 269	36.4 63.6
Marital Status	Single Married Others∗	169 225 29	40.0 53.2 6.9
Level of education	Illiterate Primary secondary Diploma BSc and above	96 73 111 68 75	22.7 17.3 26.2 16.1 17.7
Religion	Orthodox Muslim Protestant	333 85 5	78.7 20.1 1.2
Occupation	Employee Merchant Unemployed Farmer Student Housewife	91 90 30 56 128 28	21.5 21.5 7.1 13.1 30.3 6.6

∗ Divorced, Widowed.

### 4.2 Perception of respondents regarding the cause of schizophrenia

Nearly two-thirds (67.1%) and 26.2% of the respondents perceived mental illness and heredity/genetics as a cause of schizophrenia respectively. More than half of respondents believed that work stress (53.0%), unemployment (54.6%), and failure in love (54.6%) are causes of schizophrenia. From the social issue, 52.7% of the participants perceived that loneliness is the cause of schizophrenia. Nearly two-thirds (67.6%) and more than half (58.9%) of the respondents perceived that anxious personality and alcohol or other addiction are causes of schizophrenia. From the religious perspective, 54.1% of the participants perceived that the cause of schizophrenia is God's will (Table 2).

**Table 2:** Perception regarding the cause of schizophrenia among Gondar Zuria district residents, Northwest Ethiopia, 2021 (n = 423).

	Perceived cause of schizophrenia	Yes (%)	No (%)
Biological	Mental illness	284 (67.1)	139 (32.9)
	Hereditary/Genetic	111 (26.2)	312 (73.8)
Psychosocial stressor	Marital problem	170 (40.2)	254 (59.8)
	Work stress	224 (53.0)	199 (47.0)
	Busy lifestyle	306 (72.3)	117 (27.7)
	Unemployment	231 (54.6)	192 (45.4)
	Failure in love	231 (54.6)	192 (45.4)
Social issue	Loneliness	223 (52.7)	200 (47.3)
	Bad upbringing	201 (47.5)	222 (52.5)
Personality issue	Anxious personality	286 (67.6)	137 (32.4)
	Attention seeking behavior	169 (40.0)	254 (60.0)
	Alcohol or other addiction	249 (58.9)	174 (41.1)
Religious reason	Punishment for sins	150 (35.5)	273 (64.5)
	Fate	172 (40.7)	251 (59.3)
	Gods will	229 (54.1)	194 (45.9)
Superstitious beliefs	Black magic	225 (53.2)	198 (46.8)

### 4.3 Help-seeking behavior of schizophrenia among Gondar zuria district residents

Only two hundred and seventy (63.8%) of the respondents reported that they take a person with schizophrenia to a health institution (95% CI: 59.1%, 68.3%). The majority (90.8%; (95% CI: 87.6%, 93.2%)) of the respondents seek religious help. Of those, 82.7% and 84.6% reported that they pray and take to religious father/holy water if they experience individuals with schizophrenia respectively. More than half (52.5%: (95% CI; 47.7%, 57.2%)) participants responded as they would seek social interventions. From the social help perspective, getting married (32.4%), get employed (40.2%), and change a job (34.8%) are mentioned by respondents as a solution for a person with schizophrenia (Table 3).

**Table 3:** Help-seeking behavior to schizophrenia among Gondar Zuria district residents, Northwest Ethiopia, 2021 (n = 423).

	What do you do/where do you go if you face a person with schizophrenia?	Yes (%)	No (%)
Medical help	Take to health institution	270 (63.8)	153 (36.2)
Religious help	Pray	350 (82.7)	73 (17.3)
	Go to religious father/holy water	358 (84.6)	65 (15.4)
Social help	Get married	137 (32.4)	286 (67.6)
	Get employed	170 (40.2)	253 (59.8)
	Change job	147 (34.8)	276 (65.2)

### 4.4 Determinants of help-seeking behavior of schizophrenia

#### 4.4.1 Medical help-seeking behavior of schizophrenia

In Bivariable logistic regression sex, level of education, occupation, perceived biological cause, perceived social cause, perceived religious cause were variables that were competent for multivariable logistic regression. In multivariable logistic regression occupation of participants, perceived biological cause, perceived social cause, and perceived religious cause were statistically significant factors with medical help.

The odds of medical help for schizophrenia among students and unemployed were 3.43 times (AOR = 3.43; 95%CI: 1.44, 8.15) and 4.87 times (AOR = 4.87; 95%CI: 1.4, 16.40) more likely to seek medical help than farmers or housewives respectively. Those individuals who perceived the cause of schizophrenia as biological were 1.7 times (AOR = 1.7; 95%CI: 1.01, 2.89) to seek medical help than those who didn't perceive it as the biological cause. The odds of medical help among those perceived as religious cause decrease by 52% (AOR = 0.48; 95%CI: 0.29, 0.80) than those who didn't perceive it. Those Adults who perceived the cause of schizophrenia as social issues were 2.05 times (AOR = 2.05; 95%CI: 1.29, 3.25) more likely to seek medical help than those who didn't perceive (Table 4).

**Table 4:** Multivariable logistic regression of the determinant factors of medical help-seeking behavior of schizophrenia among Gondar Zuria district adults, Northwest Ethiopia, 2021 (n = 423).

Variables			Medical help		AOR (95%CI)	P-value
			Yes frequency (%)	No Frequency (%)		
Sex		Male	179 (66.54)	90 (33.46)	1.03 (0.64,1.66)	0.89
		Female	91 (59.09)	63 (40.91)	1	
Level of education		Illiterate	43 (44.79)	53 (55.21)	1	
		Primary	39 (53.42)	34 (46.58)	0.52 (0.23,1.16)	0.11
		Secondary	77 (69.37)	34 (30.63)	0.90 (0.41,1.97)	0.79
		Diploma	58 (85.29)	10 (14.71)	2.40 (0.92,6.19)	0.07
		BSc& above	53 (70.67)	22 (29.33)	0.57 (0.19,1.72)	0.32
occupation		Farmer/housewife	33 (39.29)	51 (60.71)	1	
		Employee	68 (74.73)	23 (25.27)	2.89 (0.98,8.52)	0.06
		Student	59 (74.22)	33 (25.78)	3.43 (1.44, 8.15)	<0.01∗
		Unemployed	24 (80.00)	6 (20.00)	4.87 (1.4,16.40)	0.01∗
		Merchant	50 (55.56)	40 (44.44)	1.5 (0.72,3.20)	0.27
Biological cause		Yes	211 (71.04)	86 (28.96)	1.70 (1.01,2.89)	0.05∗
		No	59 (46.83)	67 (53.17)	1	
Religious cause		Yes	151 (57.41)	112 (42.59)	0.48 (0.29,0.80)	<0.01∗
		No	119 (42.59)	41 (25.62)	1	
Social issue causes		Yes	195 (69.15)	87 (30.85)	2.05 (1.29,3.25)	<0.01∗
		No	75 (53.19)	66 (46.81)	1	

Note: ∗statistically significant at p value <0.05.

#### 4.4.2 Religious help-seeking behavior of schizophrenia

First, bivariable logistic regression was conducted and age, level of education, occupation, perceived biological cause, perceived social cause, perceived religious cause were variables that fitted for multivariable logistic regression. Level of education, occupation, and perceived religious cause were statistically significant variables of religious help-seeking behavior.

The odds of religious help among those who attended primary school decreased by 83 % (AOR = 0.17; 95%CI: 0.04, 0.76) than BSc and above holders. Those who were employed by occupation were 88% times (AOR = 0.12; 95%CI: 0.02, 0.64) less likely to seek religious help for schizophrenia than farmers or housewives. The odds of religious help-seeking for schizophrenia among those who perceived the cause of schizophrenia is religious were 2.34 times (AOR = 2.34; 95%CI: 1.06, 5.11) more likely to seek help than those who didn't perceive (Table 5).

**Table 5:** Multivariable logistic regression of the determinant factors of religious help-seeking behavior of schizophrenia among Gondar Zuria district adults, Northwest Ethiopia, 2021 (n = 423).

Variables			Religious help		AOR (95%CI)	P-value
			Yes frequency (%)	No Frequency (%)		
Age		18–24 years	140 (93.33)	10 (6.67)	3.79 (0.76,18.72)	0.10
		25–44years	184 (91.09)	18 (8.91)	2.57 (0.93,7.13)	0.06
		45 and above	60 (84.51)	11 (15.49)	1	
Level of education		Illiterate	89 (92.71)	7 (7.29	0.33 (0.06,1.62)	
		Primary	61 (83.56)	12 (16.44)	0.17 (0.04,0.76)	0.01∗
		Secondary	110 (99.10)	1 (0.90)	4.77 (0.49,46.08)	0.17
		Diploma	59 (86.76)	9 (13.24)	0.41 (0.13,1.26)	0.12
		BSc& above	65 (86.67)	10 (13.33)	1	
occupation		Farmer/housewife	79 (94.05)	5 (5.95)	1	
		Employee	75 (82.42)	16 (17.58)	0.12 (0.02,0.64)	0.01∗
		Student	119 (92.97)	9 (7.03)	0.26 (0.04,1.83)	0.18
		Unemployed	27 (90.00)	3 (10.00)	0.14 (0.02,1.08)	0.06
		Merchant	84 (93.33)	6 (6.67)	0.96 (0.23,4.01)	0.96
Biological cause		Yes	270 (90.91)	27 (9.09)	1.31 (0.55,3.04)	0.54
		No	114 (90.48)	12 (9.52)	1	
Religious cause		Yes	248 (94.30)	15 (5.70)	2.34 (1.06,5.11)	0.03∗
		No	136 (85.00)	24 (15.00)	1	
Social issue causes		Yes	254 (90.07)	28 (9.93)	0.74 (0.34,1.63)	0.34
		No	130 (92.20)	11 (7.80)	1	

Note: ∗statistically significant at p value <0.05.

#### 4.4.3 Social intervention help-seeking behavior of schizophrenia

In multivariable logistic regression age, level of education, occupation, and perceived the cause of schizophrenia as religion were statistically significant factors associated with social intervention help-seeking behavior of schizophrenia.

The odds of social intervention help-seeking for schizophrenia among those whose age group of 18–24 years old and 25–44 years old were 3.5 times (AOR = 3.5; 95% CI: 1.33, 9.18) and 1.94 times (AOR = 1.94; 95% CI: 1.03, 3.68) more likely to seek social intervention for schizophrenia than those whose age was more than or equal to 45 years old respectively (Table 6).

**Table 6:** Multivariable logistic regression of the determinant factors of social intervention help-seeking behavior of schizophrenia among Gondar Zuria district adults, Northwest Ethiopia, 2021 (n = 423).

Variables			Social interventions		AOR (95%CI)	P-value
			Yes frequency (%)	No Frequency (%)		
Age		18–24 years	101 (67.33)	49 (32.67)	3.5 (1.33, 9.18)	0.01∗
		25–44years	100 (49.50)	102 (50.50)	1.94 (1.03, 3.68)	0.04∗
		45 and above	21 (29.580)	50 (70.42)	1	
Level of education		Illiterate	32 (33.33)	64 (66.67)	0.60 (0.22, 1.66)	0.32
		Primary	43 (58.90)	30 (41.10)	1.37 (0.53, 3.56)	0.5
		Secondary	60 (54.05)	51 (45.95)	0.83 (0.35,1.96)	0.68
		Diploma	45 (66.18)	23 (33.82)	1.52 (0.69,3.35)	0.30
		BSc& above	42 (56.00)	33 (44.00)	1	1
occupation		Farmer/housewife	36 (42.86)	48 (57.14)	1.27 (0.47, 3.42)	0.62
		Employee	51 (56.04)	40 (43.96)	1	
		Student	85 (66.41)	43 (33.59)	0.92 (0.35, 2.43)	0.87
		Unemployed	18 (60.00)	12 (40.00)	0.78 (0.28, 2.15)	0.63
		Merchant	32 (35.56)	58 (64.44)	0.58 (0.25, 1.37)	0.21
Biological cause		Yes	56 (56.90)	128 (43.10)	1.31 (0.55,3.04)	0.54
		No	53 (42.06)	73 (57.94)	1	
Religious cause		Yes	134 (50.95)	129 (49.05)	0.93 (0.5, 1.46)	0.75
		No	88 (55.00)	72 (45.00)	1	
Social issue causes		Yes	157 (55.67)	125 (44.33)	0.74 (0.34,1.63)	0.34
		No	65 (46.10)	76 (53.90)	1	

Note: ∗statistically significant at p value <0.05.

## 5 Discussion

This study aimed to assess the help-seeking behavior of schizophrenia among Gondar zuria district adults. The magnitude of perceived medical help-seeking, religious help-seeking, and social intervention of schizophrenia was 63.8%, 90.8, and 52.5% respectively. Occupation, perceived biological causes, perceived religious and social issues causes of schizophrenia were statistically significant factors of perceived medical help. The statistically associated factors of perceived religious help were level of education, occupation, and perceived religious help, and being in the age group of 18–24and 25–44 was a statistically associated variable of social intervention help.

Nearly two-thirds (63.8%) of the respondents seek help from a health institution for a person with schizophrenia. This finding was relatively consistent with a study conducted in the Hunan province of China (64.6%) [1]. However, the current finding was lower than studies conducted in Mertule Mariam town, Ethiopia (81.5%), and Hunan province of China (70.4%) [2, 3]. The possible justification for this difference might be the difference in socio-demographic and local cultural factors that influence the illness-related beliefs and perceptions. It might also be due to more access to information and a higher level of awareness in the previous study that pushed them to seek medical help for schizophrenia. On the other hand, it was higher than the finding of studies conducted in China among Chinese patients (31.7%) and Pakistan (52.2%) [4, 5]. This might be due to low desire to handle the problem on one's own, high perceived need, and high mental health literacy that enables them to seek medical help for mental disorders rather than being dependent on social and religious solutions.

The result of this study revealed that the majority (90.8%) of the participants seek religious help like pray, go to a religious father, and holy water. This implies still the majority of the population perceives the intervention should be religious. The finding of this study is higher than the studies previously done at Turkey 74% [18], Hunan Province China 30.8% [19], Bilaspur India (64%), New Delhi India 56% [11], Pakistan 19.5% [5], and Mertule Mariam Ethiopia 44.6% [23]. The variation of the finding might be due to the difference in socio-demographic characteristics, sample size, and measurement tool.

The current study revealed that the magnitude of social intervention for schizophrenia among adults was 52.5%. This finding is in line with a study done in Jimma [24] higher than a study conducted in Pakistan (10.6%) and a study in Japan on help-seeking of mental illness indicated that 60% seek help from their friends which is a little higher than our finding [5, 25]. The possible justification for this variation could be the difference in sociocultural variation of the countries. Ethiopians are costumed with strong social interaction [26]. For every social event including ailments the first measurement is traditional and social interventions, they seek medical help if the traditional interventions failed. Besides a significant number of individuals perceived that the cause of schizophrenia is social issues like loneliness and bad up bring so that they might perceived these could be treated by the social interventions.

The odds of seeking medical help for schizophrenia among students and unemployed were higher than farmers or housewives. This finding was supported by a study conducted in Saudi [6]. This might be due to as an individual's educational status increases their level of understanding about mental health improved and their perception towards mentally ill will become positive. As a result, they seek help from health institutions, health professionals, and psychiatrists rather than staying at home by seeking help from traditional methods of treatment. Individuals who perceived the cause of schizophrenia as biological were nearly two times more likely to seek medical help than those who didn't perceive it as the biological cause. On the other hand, those individuals who perceived the cause of schizophrenia as a religious cause were 52% times less likely to seek medical help. This finding was supported by a study conducted in Pakistan [5]. This might be due to causal perceptions influence help-seeking behavior for schizophrenia [7]. If an individual perceived the causes of schizophrenia as biological, they might think the best solution for them is medical help and he/she will seek help from health institutions. Similarly, those who perceive the cause of schizophrenia as religious are more likely to seek help from religious institutions since they consider it as their best solution for the illness. Respondents who perceived the cause of schizophrenia as social issues were nearly two times more likely to seek medical help compared with their counterparts. This might be due to if individuals perceive schizophrenia is caused by social issues like loneliness and bad upbringing, they might seek professional medical help for counseling and advice regarding their mental illness.

Those who were employed by occupation were 88% times less likely to seek religious help for schizophrenia than farmers or housewives. It might be due to the difference in the level of education. Since employees were educated, they will have a better understanding of the cause and treatment of schizophrenia. On the other hand, employees have access to information about the cause and treatment of the disease compared with farmers. The odds of religious help seek are two times higher among peoples who perceive religion as a cause of schizophrenia compared with their counterparts. Consistent with the finding of the study conducted in Pakistan (5). It might be because those people perceive religion as the cause of schizophrenia they also perceive the treatment of religious-related remedies.

The finding of this study also showed that being in the age group of 18–24 and 25 to 44 were more likely to seek social intervention for individuals with schizophrenia. However, a study in Mertule Mariam indicated that older individuals traditional help than youngers [23]. This difference could be due to that our study was about schizophrenia only while the former study was help-seeking to common mental illness. Furthermore, the vignette case we used included that he was single so that Youngers might perceive it as a cause of his illness, and social interventions like getting married is a solution.

**This study has some limitations**. There might be an introduction of recall bias while obtaining help-seeking information for schizophrenia from the community. The study used a community-based sample, and the help-seeking behavior, as well as perceptions, may be different in a hospital-based study. The cross-sectional nature of the study does not allow a strict causal interpretation of the results. It was also better if the study was triangulated with qualitative data.

## 6 Conclusion

Nearly two-thirds, the majority, and more than half of the respondents seek medical, religious, and social help for schizophrenia respectively. This implies that the majority of the respondents seek non-medical help for the treatment of schizophrenia. Being student, unemployed, perceived biological case, and perceived social cause increases the odds of seeking medical help while perceived religious cause decreases it. Being unemployed, attending primary school decreases the odds of seeking religious help whereas perceived religious cause increases it. Young adults have higher odds of social help-seeking behavior. Young adults have higher odds of seeking social help for schizophrenia. It is better to create awareness for the community to change their perception towards the cause and treatments of schizophrenia. Integrating the religious and social interventions for schizophrenia into medical interventions is better to be considered. Besides, interventions are better to be given based on the cause of schizophrenia.

## Declarations

### Author contribution statement

Enyew Getaneh Mekonen, Ayenew Kassie Tesema, Belayneh Shetie Workneh, Maereg Wolde, Niguse Yigzaw Muluneh: Conceived and designed the experiments; Performed the experiments; Analyzed and interpreted the data; Contributed reagents, materials, analysis tools or data; Wrote the paper.

### Funding statement

This research did not receive any specific grant from funding agencies in the public, commercial, or not-for-profit sectors.

### Data availability statement

Data will be made available on request.

### Declaration of interests statement

The authors declare no conflict of interest.

### Additional information

No additional information is available for this paper.
